# Poly[[μ-aqua-aqua­[μ_4_-ethyl (dichloro­methyl­ene)diphospho­nato]sesqui­calcium(II)] acetone hemisolvate 4.5-hydrate]

**DOI:** 10.1107/S1600536809010150

**Published:** 2009-03-25

**Authors:** Jonna Jokiniemi, Sirpa Peräniemi, Jouko Vepsäläinen, Markku Ahlgrén

**Affiliations:** a Department of Chemistry, University of Joensuu, PO Box 111, FI-80101 Joensuu, Finland; b Laboratory of Chemistry, Department of Biosciences, University of Kuopio, PO Box 1627, FI-70211 Kuopio, Finland

## Abstract

The title compound, {[Ca_1.5_(C_3_H_5_Cl_2_O_6_P_2_)(H_2_O)_2_]·0.5CH_3_COCH_3_·4.5H_2_O}_*n*_, has a two-dimensional polymeric structure. The asymmetric unit contains two crystallographically independent Ca^2+^ cations connected by a chelating and bridging ethyl (dichloro­methyl­ene)diphos­pho­n­ate(3^−^) ligand and an aqua ligand. One of the Ca atoms, lying on a centre of symmetry, has a slightly distorted octa­hedral geometry, while the other Ca atom is seven-coordinated in a distorted monocapped trigonal-prismatic geometry. The polymeric layers are further connected by extensive O—H⋯O hydrogen bonding into a three-dimensional supra­molecular network. The acetone solvent mol­ecule and one uncoordin­ated water mol­ecule are located on twofold rotation axes.

## Related literature

For applications of metal complexes of bis­phospho­nates, see: Clearfield *et al.* (2001 ▶); Clearfield (1998 ▶); Fu *et al.* (2007 ▶); Serre *et al.* (2006 ▶). For calcium bis­phospho­nate complexes, see: Lin *et al.* (2007 ▶); Mathew *et al.* (1998 ▶). For metal complexes of bis­phospho­nate ester derivatives, see: Jokiniemi *et al.* (2007 ▶, 2008 ▶).
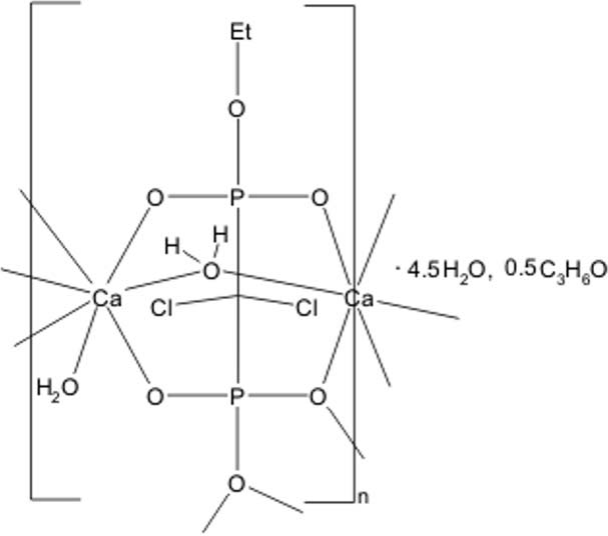


## Experimental

### 

#### Crystal data

[Ca_1.5_(C_3_H_5_Cl_2_O_6_P_2_)(H_2_O)_2_]·0.5C_3_H_6_O·4.5H_2_O
*M* *_r_* = 476.17
Monoclinic, 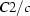

*a* = 31.2205 (3) Å
*b* = 10.1546 (1) Å
*c* = 11.6510 (1) Å
β = 103.107 (1)°
*V* = 3597.51 (6) Å^3^
*Z* = 8
Mo *K*α radiation
μ = 1.02 mm^−1^
*T* = 150 K
0.25 × 0.15 × 0.10 mm

#### Data collection

Nonius KappaCCD diffractometer
Absorption correction: multi-scan (*XPREP* in *SHELXTL*; Sheldrick, 2008 ▶) *T*_min_ = 0.823, *T*_max_ = 0.905
31118 measured reflections
4209 independent reflections
3617 reflections with *I* > 2σ(*I*)
*R*_int_ = 0.055

#### Refinement

*R*[*F* ^2^ > 2σ(*F* ^2^)] = 0.030
*wR*(*F* ^2^) = 0.073
*S* = 1.10
4209 reflections
213 parameters
H-atom parameters constrained
Δρ_max_ = 0.47 e Å^−3^
Δρ_min_ = −0.58 e Å^−3^

### 

Data collection: *COLLECT* (Nonius, 1997 ▶); cell refinement: *DENZO*/*SCALEPACK* (Otwinowski & Minor, 1997 ▶); data reduction: *DENZO*/*SCALEPACK*; program(s) used to solve structure: *SHELXS97* (Sheldrick, 2008 ▶); program(s) used to refine structure: *SHELXL97* (Sheldrick, 2008 ▶); molecular graphics: *DIAMOND* (Brandenburg, 2005 ▶); software used to prepare material for publication: *SHELXL97*.

## Supplementary Material

Crystal structure: contains datablocks I, global. DOI: 10.1107/S1600536809010150/xu2487sup1.cif

Structure factors: contains datablocks I. DOI: 10.1107/S1600536809010150/xu2487Isup2.hkl

Additional supplementary materials:  crystallographic information; 3D view; checkCIF report

## Figures and Tables

**Table 1:** Selected bond lengths (Å)

Ca1—O1	2.3778 (14)
Ca1—O11	2.2278 (14)
Ca1—O21	2.3279 (15)
Ca2—O1	2.5726 (15)
Ca2—O2	2.4024 (15)
Ca2—O11^i^	2.4049 (15)
Ca2—O12	2.3466 (14)
Ca2—O13^ii^	2.3320 (15)
Ca2—O13^i^	2.5858 (15)
Ca2—O22	2.3158 (15)

Symmetry codes: (i) 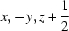; (ii) 
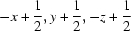
.

**Table 2:** Hydrogen-bond geometry (Å, °)

*D*—H⋯*A*	*D*—H	H⋯*A*	*D*⋯*A*	*D*—H⋯*A*
O1—H1*B*⋯O3	0.99	1.81	2.794 (2)	171
O1—H1*A*⋯O12^ii^	0.99	1.83	2.637 (2)	137
O2—H2*A*⋯O3	0.84	1.88	2.717 (2)	172
O2—H2*B*⋯O21^iii^	0.85	1.90	2.746 (2)	177
O3—H3*A*⋯O6^iv^	0.86	1.93	2.782 (2)	175
O3—H3*B*⋯O4^iii^	0.86	1.89	2.734 (2)	169
O4—H4*A*⋯O22	0.85	2.00	2.841 (2)	166
O4—H4*B*⋯O2^iv^	0.85	1.93	2.754 (2)	163
O5—H5*A*⋯O4	0.85	2.02	2.838 (2)	163
O5—H5*B*⋯O6	0.85	2.05	2.901 (3)	171
O6—H6*A*⋯O8	0.85	2.02	2.831 (2)	161
O6—H6*B*⋯O7^v^	0.84	2.26	2.832 (2)	125
O7—H7⋯O5	0.84	1.98	2.799 (2)	166

Symmetry codes: (ii) 
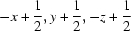
; (iii) 
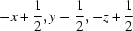
; (iv) 
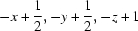
; (v) 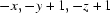.

**Fig. 1.**
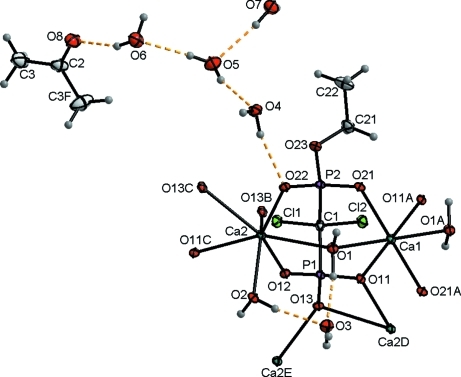
A part of the polymeric structure of the title compound showing the atomic numbering scheme and 50% probability displacement ellipsoids for non-H atoms. Hydrogen bonds are shown as dashed lines. Atoms labelled with suffixes A–F are at the symmetry positions (1/2 - x, 1/2 - y, -z), (1/2 - x, 1/2 + y, 1/2 - z), (x, -y, 1/2 + z), (x, -y, z - 1/2), (1/2 - x, y - 1/2, 1/2 - z) and (- x, y, 3/2 - z), respectively.

**Fig. 2.**
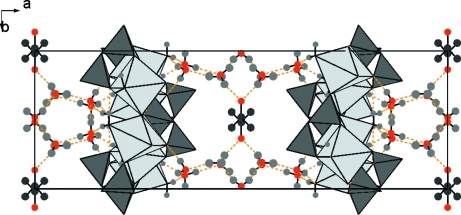
Packing of the title compound viewed along the c-axis showing the hydrogen bond interactions. CaO6 and CaO7 polyhedra are presented in light grey and PO3C tetrahedra in dark grey. Ethyl groups, chlorine atoms and H atoms of the acetone molecules are omitted for clarity.

## supplementary crystallographic information

### Comment

Metal bisphosphonates have been attracting closer attention in light of their important applications in industrial processes such as ion-exchange, catalysis and sorption (Clearfield *et al.*, 2001, Clearfield, 1998, Fu *et al.*, 2007). Metal bisphosphonates usually adopt layered or pillared layered structures (Fu *et al.*, 2007, Mathew *et al.*, 1998). Other structural types, such as 1-D and 3-D open networks, have also been prepared in order to study the properties of bisphosphonate solid materials (Lin *et al.*, 2007, Fu *et al.*, 2007). Most of the effective materials consist of open frameworks and microporous structures (Fu *et al.*, 2007, Serre *et al.*, 2006). In recent investigations, we studied the complexing properties of amide ester derivatives of (dichloromethylene)bisphosphonate, Cl_2_MBP (Jokiniemi *et al.*, 2007, 2008). The introduction of various ester substituents into phosphonate groups can results in novel structures of metal bisphosphonates and lead to interesting functionalities. Of the numerous metal phosphonate compounds now known, only a small number have been prepared with alkali earth metals. We now present the crystal structure of the Ca(II) complex of the monoethyl ester derivative of Cl_2_MBP obtained by gel crystallization.

The title compound consists of two-dimensional layers parallel to the (100) plane. The Ca1 atom lies on the centre of symmetry with two symmetrically chelating (Cl_2_CP_2_O_6_Et)^3-^ ligands and two aqua ligands in axial positions; the geometry is slightly distorted octahedron with Ca1–O bond lengths of 2.228 (1)–2.378 (1) Å (Table 1, Fig. 1). The three *trans* bond angles are 180.0°, while the *cis* bond angles range from 84.12 (5) to 95.88 (5)°. The aqua ligand O1 bridges Ca1 and the adjacent Ca2 atom with Ca···Ca distance of 4.4283 (4) Å. The Ca2 atom is seven-coordinated in distorted monocapped trigonal prismatic geometry and is coordinated by five phosphonate O atoms from three different (Cl_2_CP_2_O_6_Et)^3-^ ligands. The coordination sphere is completed by two aqua ligands. The Ca2–O bond lengths are 2.316 (2)–2.586 (2) Å. The (Cl_2_CP_2_O_6_Et)^3-^ ligand is coordinated to four Ca^2+^ cations through five O atoms forming two six-membered chelate rings with Ca1 and Ca2 atoms, and the P1 atom forms a four-membered chelate ring with the adjacent Ca2D atom (*x*, -*y*, *z* - 1/2). Thus, the two oxygen atoms (O11, O13) act as monoatomic bridges between two Ca atoms.

The layers are further connected by extensive hydrogen bonding (O···O 2.637 (2)–2.901 (3) Å, 125–177°) into a 3-D network with the interlayer distance of 15.2036 (2) Å (Fig. 2, Table 2). The O8 and C2 atoms of the acetone molecule, as well as the water molecule O7, are located on the individual two-fold rotation axis. The ethyl groups and chlorine atoms point out from the layers.

### Experimental

Na_3_Cl_2_CP_2_O_6_Et (10.0 mg, 0.030 mmol) and CaCl_2_.2H_2_O (4.3 mg, 0.030 mmol) were dissolved separately in water (2.25 ml), the solutions were mixed, and tetramethoxysilane (TMOS 0.5 ml) was added. The two-phase system was shaken until homogeneous. After gel formation, a precipitant, acetone (1.0 ml), was added above the gel to induce crystallization. After about three months, colourless crystals suitable for X-ray analysis were formed uniformly throughout the gel as thin needles. The elemental analyses were performed several times and the results were consistent indicating that the acetone molecule and 3.5 water molecules were evaporated when the crystals were dried in air.

### Refinement

H atoms of the ethyl group and acetone molecule were placed at calculated positions in the riding-model approximation with C–H distances of 0.99 Å (methylene) and 0.98 Å (methyl), and with *U*_iso_(H) = 1.5*U*_eq_(C) or 1.2*U*_eq_(C). H atoms of the aqua ligands and lattice water molecules were located in a difference map and treated as riding, with O–H bond lengths constrained to 0.84–0.99 Å and with *U*_iso_(H) = 1.5*U*_eq_(O) or 1.2*U*_eq_(O).

### Crystal data

**Table:**

[Ca_1.5_(C_3_H_5_Cl_2_O_6_P_2_)(H_2_O)_2_]·0.5C_3_H_6_O·4.5H_2_O	*F*(000) = 1968
*M**_r_* = 476.17	*D*_x_ = 1.758 Mg m^−^^3^
Monoclinic, *C*2/*c*	Mo *K*α radiation, λ = 0.71073 Å
Hall symbol: -C 2yc	Cell parameters from 31118 reflections
*a* = 31.2205 (3) Å	θ = 2.7–28.0°
*b* = 10.1546 (1) Å	µ = 1.02 mm^−^^1^
*c* = 11.6510 (1) Å	*T* = 150 K
β = 103.107 (1)°	Needle, colourless
*V* = 3597.51 (6) Å^3^	0.25 × 0.15 × 0.10 mm
*Z* = 8	

### Data collection

**Table:**

Nonius KappaCCD diffractometer	4209 independent reflections
Radiation source: fine-focus sealed tube	3617 reflections with *I* > 2σ(*I*)
graphite	*R*_int_ = 0.055
φ scans, and ω scans with κ offsets	θ_max_ = 28.0°, θ_min_ = 2.7°
Absorption correction: multi-scan (*XPREP* in *SHELXTL*; Sheldrick, 2008)	*h* = −40→40
*T*_min_ = 0.823, *T*_max_ = 0.905	*k* = −13→13
31118 measured reflections	*l* = −14→15

### Refinement

**Table:**

Refinement on *F*^2^	Primary atom site location: structure-invariant direct methods
Least-squares matrix: full	Secondary atom site location: difference Fourier map
*R*[*F*^2^ > 2σ(*F*^2^)] = 0.030	Hydrogen site location: inferred from neighbouring sites
*wR*(*F*^2^) = 0.073	H-atom parameters constrained
*S* = 1.10	*w* = 1/[σ^2^(*F*_o_^2^) + (0.02*P*)^2^ + 12*P*] where *P* = (*F*_o_^2^ + 2*F*_c_^2^)/3
4209 reflections	(Δ/σ)_max_ = 0.001
213 parameters	Δρ_max_ = 0.47 e Å^−^^3^
0 restraints	Δρ_min_ = −0.58 e Å^−^^3^

### Special details

**Table:**

Experimental. These results are supported by the IR spectrum and TG analysis. Anal. Found: C, 9.30; H, 3.06%. Calc. for C_3_H_11_Cl_2_Ca_1.5_O_9_P_2_: C, 9.38; H, 2.89%. Main IR absorptions (KBr pellet, cm^-1^): 3385 (b,s), 2995 (w), 1648 (b,m), 1389 (*m*), 1213 (*s*), 1148 (*s*), 1105 (*versus*), 1082 (*versus*), 1048 (*m*), 1008 (*m*), 959 (*m*), 871 (*m*), 852 (w), 760 (*m*). ^31^P CP/MAS NMR: δ_P_ 7.4 and 5.1 p.p.m.. TGA (25–700 °C under a synthetic air): 25–180 °C 13.1% (calculated 14.1% for the loss of three water molecules). The observed total weight loss is 40.0% (calculated 41.1% if the final product is assumed to be a mixture of Ca(PO_3_)_2_ and CaO in a molar ratio of 2:1).
Geometry. All e.s.d.'s (except the e.s.d. in the dihedral angle between two l.s. planes) are estimated using the full covariance matrix. The cell e.s.d.'s are taken into account individually in the estimation of e.s.d.'s in distances, angles and torsion angles; correlations between e.s.d.'s in cell parameters are only used when they are defined by crystal symmetry. An approximate (isotropic) treatment of cell e.s.d.'s is used for estimating e.s.d.'s involving l.s. planes.
Refinement. Refinement of *F*^2^ against ALL reflections. The weighted *R*-factor *wR* and goodness of fit *S* are based on *F*^2^, conventional *R*-factors *R* are based on *F*, with *F* set to zero for negative *F*^2^. The threshold expression of *F*^2^ > σ(*F*^2^) is used only for calculating *R*-factors(gt) *etc*. and is not relevant to the choice of reflections for refinement. *R*-factors based on *F*^2^ are statistically about twice as large as those based on *F*, and *R*- factors based on ALL data will be even larger.

### Fractional atomic coordinates and isotropic or equivalent isotropic displacement parameters (Å^2^)

**Table:**

	*x*	*y*	*z*	*U*_iso_*/*U*_eq_	
Ca1	0.2500	0.2500	0.0000	0.00999 (12)	
Ca2	0.248223 (14)	0.12782 (4)	0.36375 (3)	0.00930 (9)	
Cl1	0.129792 (17)	−0.08747 (5)	0.13650 (4)	0.01375 (11)	
Cl2	0.137474 (18)	0.01918 (5)	−0.08762 (4)	0.01548 (11)	
P1	0.218448 (17)	−0.04904 (5)	0.08794 (4)	0.00900 (11)	
P2	0.160183 (17)	0.18410 (5)	0.12307 (5)	0.00960 (11)	
O1	0.28281 (5)	0.23098 (14)	0.20444 (12)	0.0126 (3)	
H1A	0.2911	0.3217	0.2318	0.015*	
H1B	0.3108	0.1830	0.2091	0.015*	
O2	0.32098 (5)	0.03608 (14)	0.41612 (13)	0.0129 (3)	
H2A	0.3353	0.0476	0.3639	0.019*	
H2B	0.3198	−0.0467	0.4244	0.019*	
O3	0.36311 (5)	0.09774 (16)	0.24321 (14)	0.0178 (3)	
H3A	0.3899	0.1215	0.2683	0.027*	
H3B	0.3643	0.0198	0.2163	0.027*	
O4	0.13454 (5)	0.36331 (16)	0.37057 (14)	0.0180 (3)	
H4A	0.1472	0.3000	0.3430	0.027*	
H4B	0.1530	0.3864	0.4332	0.027*	
O5	0.05044 (6)	0.30916 (18)	0.41409 (16)	0.0269 (4)	
H5A	0.0775	0.3154	0.4142	0.040*	
H5B	0.0476	0.3118	0.4851	0.040*	
O6	0.05040 (6)	0.32700 (17)	0.66254 (16)	0.0238 (4)	
H6A	0.0405	0.2579	0.6883	0.036*	
H6B	0.0285	0.3765	0.6374	0.036*	
O7	0.0000	0.4816 (2)	0.2500	0.0224 (5)	
H7	0.0189	0.4385	0.2985	0.034*	
O8	0.0000	0.1357 (2)	0.7500	0.0265 (6)	
O11	0.24121 (5)	0.03290 (14)	0.00999 (12)	0.0107 (3)	
O12	0.23607 (5)	−0.03674 (14)	0.21857 (12)	0.0108 (3)	
O13	0.21597 (5)	−0.18636 (14)	0.04307 (12)	0.0103 (3)	
O21	0.18181 (5)	0.26929 (14)	0.04781 (13)	0.0116 (3)	
O22	0.17957 (5)	0.18469 (14)	0.25168 (12)	0.0115 (3)	
O23	0.10987 (5)	0.21652 (15)	0.10779 (13)	0.0132 (3)	
C1	0.16168 (7)	0.0176 (2)	0.06555 (17)	0.0104 (4)	
C21	0.08235 (8)	0.2669 (2)	0.0003 (2)	0.0196 (5)	
H21A	0.0615	0.1982	−0.0384	0.024*	
H21B	0.1006	0.2944	−0.0548	0.024*	
C22	0.05800 (9)	0.3813 (3)	0.0325 (3)	0.0292 (6)	
H22A	0.0416	0.3541	0.0911	0.044*	
H22B	0.0374	0.4143	−0.0380	0.044*	
H22C	0.0788	0.4511	0.0656	0.044*	
C2	0.0000	0.0182 (3)	0.7500	0.0228 (7)	
C3	−0.02303 (10)	−0.0568 (3)	0.8273 (3)	0.0394 (7)	
H3C	−0.0317	0.0033	0.8837	0.059*	
H3D	−0.0033	−0.1244	0.8701	0.059*	
H3E	−0.0493	−0.0990	0.7791	0.059*	

### Atomic displacement parameters (Å^2^)

**Table:**

	*U*^11^	*U*^22^	*U*^33^	*U*^12^	*U*^13^	*U*^23^
Ca1	0.0143 (3)	0.0066 (3)	0.0097 (3)	−0.0003 (2)	0.0041 (2)	0.0006 (2)
Ca2	0.0130 (2)	0.00660 (18)	0.00840 (19)	−0.00024 (15)	0.00261 (15)	0.00003 (14)
Cl1	0.0156 (2)	0.0112 (2)	0.0157 (3)	−0.00233 (19)	0.00603 (19)	0.00042 (18)
Cl2	0.0205 (3)	0.0151 (2)	0.0094 (2)	0.0024 (2)	0.00041 (19)	−0.00142 (18)
P1	0.0129 (3)	0.0059 (2)	0.0088 (3)	0.00034 (19)	0.00368 (19)	0.00012 (18)
P2	0.0122 (3)	0.0072 (2)	0.0097 (3)	0.00102 (19)	0.0030 (2)	0.00008 (18)
O1	0.0177 (8)	0.0088 (7)	0.0112 (7)	−0.0003 (6)	0.0030 (6)	−0.0011 (5)
O2	0.0160 (7)	0.0083 (7)	0.0154 (8)	−0.0001 (6)	0.0054 (6)	0.0010 (6)
O3	0.0171 (8)	0.0164 (8)	0.0199 (8)	0.0005 (6)	0.0045 (6)	0.0000 (6)
O4	0.0205 (8)	0.0176 (8)	0.0152 (8)	0.0029 (6)	0.0027 (6)	−0.0036 (6)
O5	0.0227 (9)	0.0291 (10)	0.0296 (10)	−0.0008 (8)	0.0074 (7)	−0.0014 (8)
O6	0.0200 (9)	0.0214 (9)	0.0297 (10)	−0.0012 (7)	0.0053 (7)	0.0032 (7)
O7	0.0205 (12)	0.0243 (13)	0.0216 (12)	0.000	0.0032 (10)	0.000
O8	0.0306 (14)	0.0174 (12)	0.0344 (15)	0.000	0.0130 (11)	0.000
O11	0.0159 (7)	0.0068 (7)	0.0109 (7)	0.0000 (6)	0.0057 (6)	0.0007 (5)
O12	0.0146 (7)	0.0086 (7)	0.0092 (7)	0.0005 (6)	0.0027 (6)	−0.0010 (5)
O13	0.0137 (7)	0.0072 (7)	0.0103 (7)	0.0009 (5)	0.0030 (6)	−0.0011 (5)
O21	0.0155 (7)	0.0068 (7)	0.0135 (7)	0.0009 (6)	0.0052 (6)	0.0003 (5)
O22	0.0146 (7)	0.0095 (7)	0.0102 (7)	0.0017 (6)	0.0025 (6)	−0.0010 (5)
O23	0.0130 (7)	0.0128 (7)	0.0138 (7)	0.0040 (6)	0.0031 (6)	0.0019 (6)
C1	0.0134 (10)	0.0085 (9)	0.0095 (10)	−0.0008 (8)	0.0031 (8)	0.0006 (7)
C21	0.0190 (11)	0.0221 (12)	0.0155 (11)	0.0052 (9)	−0.0009 (9)	0.0015 (9)
C22	0.0262 (13)	0.0223 (13)	0.0356 (15)	0.0100 (11)	−0.0002 (11)	0.0030 (11)
C2	0.0166 (16)	0.0189 (17)	0.033 (2)	0.000	0.0050 (14)	0.000
C3	0.0362 (16)	0.0276 (15)	0.061 (2)	0.0062 (13)	0.0253 (15)	0.0149 (14)

### Geometric parameters (Å, °)

**Table:**

Ca1—O1^i^	2.3778 (14)	P2—O22	1.4834 (15)
Ca1—O1	2.3778 (14)	P2—O21	1.4972 (15)
Ca1—O11	2.2278 (14)	P2—O23	1.5750 (15)
Ca1—O11^i^	2.2278 (14)	P2—C1	1.823 (2)
Ca1—O21^i^	2.3279 (15)	O1—H1A	0.9900
Ca1—O21	2.3279 (15)	O1—H1B	0.9900
Ca1—P2^i^	3.4915 (5)	O2—H2A	0.8414
Ca1—P2	3.4915 (5)	O2—H2B	0.8477
Ca1—P1^i^	3.4204 (5)	O3—H3A	0.8560
Ca1—P1	3.4204 (5)	O3—H3B	0.8554
Ca1—Ca2^ii^	4.1476 (4)	O4—H4A	0.8541
Ca1—Ca2^iii^	4.1476 (4)	O4—H4B	0.8536
Ca2—O1	2.5726 (15)	O5—H5A	0.8468
Ca2—O2	2.4024 (15)	O5—H5B	0.8525
Ca2—O11^iv^	2.4049 (15)	O6—H6A	0.8481
Ca2—O12	2.3466 (14)	O6—H6B	0.8448
Ca2—O13^iii^	2.3320 (15)	O7—H7	0.8416
Ca2—O13^iv^	2.5858 (15)	O8—C2	1.193 (4)
Ca2—O22	2.3158 (15)	O11—Ca2^ii^	2.4049 (15)
Ca2—P1^iv^	3.0705 (6)	O13—Ca2^vi^	2.3320 (15)
Ca2—P1^iii^	3.4498 (6)	O13—Ca2^ii^	2.5858 (15)
Ca2—P2	3.4999 (7)	O23—C21	1.442 (3)
Ca2—Ca2^v^	4.0111 (8)	C21—C22	1.482 (3)
Ca2—Ca1^vi^	4.1476 (4)	C21—H21A	0.9900
Ca2—H2A	2.8382	C21—H21B	0.9900
Ca2—H2B	2.8142	C22—H22A	0.9800
Cl1—C1	1.785 (2)	C22—H22B	0.9800
Cl2—C1	1.773 (2)	C22—H22C	0.9800
P1—O13	1.4851 (15)	C2—C3^vii^	1.484 (3)
P1—O12	1.5016 (15)	C2—C3	1.484 (3)
P1—O11	1.5216 (15)	C3—H3C	0.9800
P1—C1	1.860 (2)	C3—H3D	0.9800
P1—Ca2^ii^	3.0705 (6)	C3—H3E	0.9800
P1—Ca2^vi^	3.4498 (6)		
			
O21^i^—Ca1—O21	180.00 (6)	P2—Ca2—Ca2^v^	114.135 (18)
O21^i^—Ca1—O11	93.40 (5)	O22—Ca2—Ca1^vi^	112.14 (4)
O21—Ca1—O11	86.60 (5)	O13^iii^—Ca2—Ca1^vi^	127.37 (4)
O21^i^—Ca1—O11^i^	86.60 (5)	O2—Ca2—Ca1^vi^	67.31 (4)
O21—Ca1—O11^i^	93.40 (5)	O12—Ca2—Ca1^vi^	66.62 (4)
O11—Ca1—O11^i^	180.00 (8)	O11^iv^—Ca2—Ca1^vi^	25.39 (3)
O21^i^—Ca1—O1^i^	88.67 (5)	O1—Ca2—Ca1^vi^	132.96 (4)
O21—Ca1—O1^i^	91.33 (5)	O13^iv^—Ca2—Ca1^vi^	82.95 (3)
O11—Ca1—O1^i^	95.88 (5)	P1^iv^—Ca2—Ca1^vi^	54.110 (11)
O11^i^—Ca1—O1^i^	84.12 (5)	P1^iii^—Ca2—Ca1^vi^	147.146 (14)
O21^i^—Ca1—O1	91.33 (5)	P2—Ca2—Ca1^vi^	113.429 (13)
O21—Ca1—O1	88.67 (5)	Ca2^v^—Ca2—Ca1^vi^	105.887 (14)
O11—Ca1—O1	84.12 (5)	O22—Ca2—H2A	146.7
O11^i^—Ca1—O1	95.88 (5)	O13^iii^—Ca2—H2A	82.8
O1^i^—Ca1—O1	180.00 (11)	O2—Ca2—H2A	15.8
O21^i^—Ca1—P2^i^	19.02 (4)	O12—Ca2—H2A	78.1
O21—Ca1—P2^i^	160.98 (4)	O11^iv^—Ca2—H2A	92.6
O11—Ca1—P2^i^	109.33 (4)	O1—Ca2—H2A	63.8
O11^i^—Ca1—P2^i^	70.67 (4)	O13^iv^—Ca2—H2A	127.9
O1^i^—Ca1—P2^i^	77.10 (4)	P1^iv^—Ca2—H2A	113.6
O1—Ca1—P2^i^	102.90 (4)	P1^iii^—Ca2—H2A	91.1
O21^i^—Ca1—P2	160.98 (4)	P2—Ca2—H2A	128.7
O21—Ca1—P2	19.02 (4)	Ca2^v^—Ca2—H2A	108.8
O11—Ca1—P2	70.67 (4)	Ca1^vi^—Ca2—H2A	78.7
O11^i^—Ca1—P2	109.33 (4)	O22—Ca2—H2B	151.6
O1^i^—Ca1—P2	102.90 (4)	O13^iii^—Ca2—H2B	97.1
O1—Ca1—P2	77.10 (4)	O2—Ca2—H2B	16.4
P2^i^—Ca1—P2	180.000 (18)	O12—Ca2—H2B	73.8
O21^i^—Ca1—P1^i^	70.24 (4)	O11^iv^—Ca2—H2B	65.8
O21—Ca1—P1^i^	109.76 (4)	O1—Ca2—H2B	89.9
O11—Ca1—P1^i^	160.29 (4)	O13^iv^—Ca2—H2B	112.0
O11^i^—Ca1—P1^i^	19.71 (4)	P1^iv^—Ca2—H2B	89.9
O1^i^—Ca1—P1^i^	73.49 (4)	P1^iii^—Ca2—H2B	111.4
O1—Ca1—P1^i^	106.51 (4)	P2—Ca2—H2B	137.6
P2^i^—Ca1—P1^i^	52.709 (12)	Ca2^v^—Ca2—H2B	108.2
P2—Ca1—P1^i^	127.291 (12)	Ca1^vi^—Ca2—H2B	51.7
O21^i^—Ca1—P1	109.76 (4)	H2A—Ca2—H2B	27.5
O21—Ca1—P1	70.24 (4)	O13—P1—O12	114.38 (8)
O11—Ca1—P1	19.71 (4)	O13—P1—O11	107.30 (8)
O11^i^—Ca1—P1	160.29 (4)	O12—P1—O11	116.49 (8)
O1^i^—Ca1—P1	106.51 (4)	O13—P1—C1	108.70 (9)
O1—Ca1—P1	73.49 (4)	O12—P1—C1	103.32 (9)
P2^i^—Ca1—P1	127.291 (12)	O11—P1—C1	106.05 (9)
P2—Ca1—P1	52.709 (12)	O13—P1—Ca2^ii^	57.15 (6)
P1^i^—Ca1—P1	180.000 (17)	O12—P1—Ca2^ii^	140.52 (6)
O21^i^—Ca1—Ca2^ii^	76.36 (4)	O11—P1—Ca2^ii^	50.37 (6)
O21—Ca1—Ca2^ii^	103.64 (4)	C1—P1—Ca2^ii^	116.00 (7)
O11—Ca1—Ca2^ii^	27.57 (4)	O12—P1—Ca2^vi^	83.46 (6)
O11^i^—Ca1—Ca2^ii^	152.43 (4)	O11—P1—Ca2^vi^	116.94 (6)
O1^i^—Ca1—Ca2^ii^	74.12 (4)	C1—P1—Ca2^vi^	127.72 (7)
O1—Ca1—Ca2^ii^	105.88 (4)	Ca2^ii^—P1—Ca2^vi^	75.679 (16)
P2^i^—Ca1—Ca2^ii^	87.845 (10)	O13—P1—Ca1	136.36 (6)
P2—Ca1—Ca2^ii^	92.155 (10)	O12—P1—Ca1	99.53 (6)
P1^i^—Ca1—Ca2^ii^	133.342 (10)	C1—P1—Ca1	87.98 (6)
P1—Ca1—Ca2^ii^	46.658 (10)	Ca2^ii^—P1—Ca1	79.232 (14)
O21^i^—Ca1—Ca2^iii^	103.64 (4)	Ca2^vi^—P1—Ca1	142.808 (18)
O21—Ca1—Ca2^iii^	76.36 (4)	O22—P2—O21	117.03 (9)
O11—Ca1—Ca2^iii^	152.43 (4)	O22—P2—O23	106.35 (8)
O11^i^—Ca1—Ca2^iii^	27.57 (4)	O21—P2—O23	112.56 (8)
O1^i^—Ca1—Ca2^iii^	105.88 (4)	O22—P2—C1	109.66 (9)
O1—Ca1—Ca2^iii^	74.12 (4)	O21—P2—C1	105.54 (9)
P2^i^—Ca1—Ca2^iii^	92.155 (10)	O23—P2—C1	105.09 (9)
P2—Ca1—Ca2^iii^	87.845 (10)	O22—P2—Ca1	103.43 (6)
P1^i^—Ca1—Ca2^iii^	46.658 (10)	O23—P2—Ca1	141.89 (6)
P1—Ca1—Ca2^iii^	133.342 (10)	C1—P2—Ca1	86.38 (7)
Ca2^ii^—Ca1—Ca2^iii^	180.000 (14)	O21—P2—Ca2	100.78 (6)
O22—Ca2—O13^iii^	110.21 (5)	O23—P2—Ca2	134.96 (6)
O22—Ca2—O2	160.19 (5)	C1—P2—Ca2	93.57 (7)
O13^iii^—Ca2—O2	82.51 (5)	Ca1—P2—Ca2	78.603 (13)
O22—Ca2—O12	78.08 (5)	Ca1—O1—Ca2	126.86 (6)
O13^iii^—Ca2—O12	153.36 (5)	Ca1—O1—H1A	105.6
O2—Ca2—O12	84.02 (5)	Ca2—O1—H1A	105.6
O22—Ca2—O11^iv^	110.33 (5)	Ca1—O1—H1B	105.6
O13^iii^—Ca2—O11^iv^	109.29 (5)	Ca2—O1—H1B	105.6
O2—Ca2—O11^iv^	77.85 (5)	H1A—O1—H1B	106.1
O12—Ca2—O11^iv^	90.09 (5)	Ca2—O2—H2A	112.9
O22—Ca2—O1	88.74 (5)	Ca2—O2—H2B	110.5
O13^iii^—Ca2—O1	76.76 (5)	H2A—O2—H2B	105.3
O2—Ca2—O1	79.26 (5)	H3A—O3—H3B	105.4
O12—Ca2—O1	78.20 (5)	H4A—O4—H4B	104.4
O11^iv^—Ca2—O1	155.24 (5)	H5A—O5—H5B	108.6
O22—Ca2—O13^iv^	85.30 (5)	H6A—O6—H6B	106.6
O13^iii^—Ca2—O13^iv^	70.81 (6)	P1—O11—Ca1	130.70 (8)
O2—Ca2—O13^iv^	113.76 (5)	P1—O11—Ca2^ii^	100.46 (7)
O12—Ca2—O13^iv^	135.83 (5)	Ca1—O11—Ca2^ii^	127.05 (6)
O11^iv^—Ca2—O13^iv^	57.92 (5)	P1—O12—Ca2	138.76 (9)
O1—Ca2—O13^iv^	142.51 (5)	P1—O13—Ca2^vi^	127.93 (8)
O22—Ca2—P1^iv^	97.16 (4)	P1—O13—Ca2^ii^	94.01 (7)
O13^iii^—Ca2—P1^iv^	91.17 (4)	Ca2^vi^—O13—Ca2^ii^	109.19 (6)
O2—Ca2—P1^iv^	97.71 (4)	P2—O21—Ca1	130.53 (8)
O12—Ca2—P1^iv^	113.39 (4)	P2—O22—Ca2	133.01 (9)
O11^iv^—Ca2—P1^iv^	29.16 (3)	C21—O23—P2	123.71 (14)
O1—Ca2—P1^iv^	167.82 (4)	Cl2—C1—Cl1	108.43 (11)
O13^iv^—Ca2—P1^iv^	28.85 (3)	Cl2—C1—P1	108.63 (11)
O22—Ca2—P1^iii^	93.51 (4)	Cl1—C1—P1	109.34 (11)
O13^iii^—Ca2—P1^iii^	19.85 (4)	Cl2—C1—P2	108.74 (11)
O2—Ca2—P1^iii^	95.43 (4)	Cl1—C1—P2	108.71 (11)
O12—Ca2—P1^iii^	142.04 (4)	P1—C1—P2	112.90 (11)
O11^iv^—Ca2—P1^iii^	127.05 (4)	O23—C21—C22	107.30 (19)
O1—Ca2—P1^iii^	64.53 (3)	O23—C21—H21A	110.3
O13^iv^—Ca2—P1^iii^	78.92 (3)	C22—C21—H21A	110.3
P1^iv^—Ca2—P1^iii^	104.321 (16)	O23—C21—H21B	110.3
O22—Ca2—P2	18.06 (4)	C22—C21—H21B	110.3
O13^iii^—Ca2—P2	116.57 (4)	H21A—C21—H21B	108.5
O2—Ca2—P2	142.41 (4)	C21—C22—H22A	109.5
O12—Ca2—P2	64.50 (4)	C21—C22—H22B	109.5
O11^iv^—Ca2—P2	119.83 (4)	H22A—C22—H22B	109.5
O1—Ca2—P2	74.77 (4)	C21—C22—H22C	109.5
O13^iv^—Ca2—P2	103.35 (3)	H22A—C22—H22C	109.5
P1^iv^—Ca2—P2	112.947 (18)	H22B—C22—H22C	109.5
P1^iii^—Ca2—P2	97.378 (15)	O8—C2—C3^vii^	120.89 (17)
O22—Ca2—Ca2^v^	98.51 (4)	O8—C2—C3	120.89 (17)
O13^iii^—Ca2—Ca2^v^	37.50 (4)	C3^vii^—C2—C3	118.2 (3)
O2—Ca2—Ca2^v^	100.60 (4)	C2—C3—H3C	109.5
O12—Ca2—Ca2^v^	169.12 (4)	C2—C3—H3D	109.5
O11^iv^—Ca2—Ca2^v^	81.36 (4)	H3C—C3—H3D	109.5
O1—Ca2—Ca2^v^	112.23 (4)	C2—C3—H3E	109.5
O13^iv^—Ca2—Ca2^v^	33.30 (3)	H3C—C3—H3E	109.5
P1^iv^—Ca2—Ca2^v^	56.443 (13)	H3D—C3—H3E	109.5
P1^iii^—Ca2—Ca2^v^	47.877 (12)		

Symmetry codes: (i) −*x*+1/2, −*y*+1/2, −*z*; (ii) *x*, −*y*, *z*−1/2; (iii) −*x*+1/2, *y*+1/2, −*z*+1/2; (iv) *x*, −*y*, *z*+1/2; (v) −*x*+1/2, −*y*+1/2, −*z*+1; (vi) −*x*+1/2, *y*−1/2, −*z*+1/2; (vii) −*x*, *y*, −*z*+3/2.

### Hydrogen-bond geometry (Å, °)

**Table:**

*D*—H···*A*	*D*—H	H···*A*	*D*···*A*	*D*—H···*A*
O1—H1B···O3	0.99	1.81	2.794 (2)	171
O1—H1A···O12^iii^	0.99	1.83	2.637 (2)	137
O2—H2A···O3	0.84	1.88	2.717 (2)	172
O2—H2B···O21^vi^	0.85	1.90	2.746 (2)	177
O3—H3A···O6^v^	0.86	1.93	2.782 (2)	175
O3—H3B···O4^vi^	0.86	1.89	2.734 (2)	169
O4—H4A···O22	0.85	2.00	2.841 (2)	166
O4—H4B···O2^v^	0.85	1.93	2.754 (2)	163
O5—H5A···O4	0.85	2.02	2.838 (2)	163
O5—H5B···O6	0.85	2.05	2.901 (3)	171
O6—H6A···O8	0.85	2.02	2.831 (2)	161
O6—H6B···O7^viii^	0.84	2.26	2.832 (2)	125
O7—H7···O5	0.84	1.98	2.799 (2)	166

Symmetry codes: (iii) −*x*+1/2, *y*+1/2, −*z*+1/2; (vi) −*x*+1/2, *y*−1/2, −*z*+1/2; (v) −*x*+1/2, −*y*+1/2, −*z*+1; (viii) −*x*, −*y*+1, −*z*+1.
